# “I can't let it stop me”: perspectives on aging from older adults with sickle cell disease

**DOI:** 10.1093/jscdis/yoaf017

**Published:** 2025-04-25

**Authors:** Charity I Oyedeji, Tolu O Oyesanya, Rania Mohamed, Stephanie Padrick, Reena Ravi, Teagan Callaway, John J Strouse

**Affiliations:** Department of Medicine, Division of Hematology, Duke University School of Medicine, Durham, NC 27710, United States; Claude D. Pepper Older Americans Independence Center, Duke University, Durham, NC 27710, United States; Duke Comprehensive Sickle Cell Center, Duke University School of Medicine, Durham, NC 27710, United States; School of Nursing, Duke University, Durham, NC 27710, United States; Department of Medicine, Division of Hematology, Duke University School of Medicine, Durham, NC 27710, United States; Department of Medicine, Division of Hematology, Duke University School of Medicine, Durham, NC 27710, United States; Department of Medicine, Division of Hematology, Duke University School of Medicine, Durham, NC 27710, United States; Department of Medicine, Division of Hematology, Duke University School of Medicine, Durham, NC 27710, United States; Department of Medicine, Division of Hematology, Duke University School of Medicine, Durham, NC 27710, United States; Claude D. Pepper Older Americans Independence Center, Duke University, Durham, NC 27710, United States; Duke Comprehensive Sickle Cell Center, Duke University School of Medicine, Durham, NC 27710, United States; Division of Pediatric Hematology/Oncology, Duke University, Durham, NC 27710, United States

**Keywords:** SCA, Hb SC disease, qualitative research, survival, quality of life

## Abstract

**Objectives:**

More individuals with SCD are living beyond initial life expectancy. Despite a growing population of older adults with SCD, little is known about their unique experiences and needs. Understanding the perspectives of older adults with SCD (age ≥ 50 years) could provide insight on the most pressing concerns that healthcare providers should focus on and strategies to promote healthy aging. The purpose of this study was to describe the aging experiences of older adults with SCD.

**Methods:**

In this qualitative descriptive study, we conducted semi-structured interviews with 19 older adults with SCD who received care at a single comprehensive sickle cell program in the Southeastern United States. Data were analyzed using conventional content analysis.

**Results:**

A total of 3 themes were identified. Theme 1 was “challenges with aging” with 2 subthemes: (a) internal challenges and (b) external challenges. Theme 2 was “wisdom gained with age for prevention and management of complications” with 2 subthemes: (a) lifestyle modifications and preventing complications and (b) managing sickle cell pain. Theme 3 was “living beyond life expectancy” with 2 subthemes: (a) differences in expectations for life expectancy and (b) factors contributing to longevity.

**Conclusion:**

These perspectives from older adults with SCD provide guidance for healthcare providers on areas that are most important to them as they age. This also provides practical strategies for prevention and self-management of SCD complications that our participants reported contributed to their quality of life and longevity.

## Teaser

As the largest study to date describing the unique lived experiences of older men and women living with SCD, this qualitative descriptive study enriches the understanding of the biggest problems this population faces. The findings of this study provide deep insights into strategies participants felt helped them to live beyond life expectancy, such as their faith, staying on top of their self-care, and healthy lifestyle choices. Older adults with SCD in this study described high levels of self-care abilities and self-efficacy. The wisdom they have gained over the years can be shared with younger adults with SCD promote healthy aging.

## INTRODUCTION

Advances in the management of individuals with SCD has led to an increase in median life expectancy from 14.3 years in 1973 to 40 to 45 years in population-based studies and 61 years in recent cohorts from comprehensive SCD programs.^1–4^ Despite improvements in life expectancy, there are minimal data on the unique aging experiences of older adults with SCD. As the population of older adults with SCD grows, there is a critical need to develop strategies to address both sickle cell and age-related needs in this population.

Older adults with SCD experience chronic sickle cell complications in addition to complications related to physiologic aging.^5^^,^^6^ Compared to younger adults (age 18-49) with SCD, older adults (age ≥ 50) with SCD are more likely to experience renal and cardiopulmonary disease, avascular necrosis requiring joint replacement, leg ulcers, and retinopathy.^7^ Older adults with SCD also experience accelerated aging and functional decline with physical performance similar to individuals in the general population who are 20-30 years older.^8–10^ Complications of physiologic aging can further exacerbate SCD-related comorbidities. Despite having more chronic complications and a similar proportion of individuals requiring daily opioids, older adults with SCD have fewer hospitalizations compared to younger adults with SCD.^7^

Although several investigators have described the clinical differences between younger and older adults with SCD, the experiences of older adults with SCD have not been well-described.^7^^,^^11–13^ One prior qualitative study interviewed 6 older women with SCD (mean age 54; range 48-64 years) on successful aging.^14^ They described 3 themes including (1) vulnerability factors such as pain crises, overprotection from parents, and limitations imposed by others and caused by SCD; (2) self-care management resources such as religion/spirituality, assertiveness when accessing healthcare, social support, and self-care activities; and (3) health outcomes including living beyond expectations and enjoying life satisfactions of having children and working.^14^ While this study helped to improve our understanding of health-related experiences for older adults with SCD, the study only included a small sample of women. We remain without data on modern challenges older men and women with SCD face as they age and current strategies they use to cope with these challenges.

To address this gap in knowledge, additional research that further describes the experiences of both older men and women with SCD is needed. The objective of this study was to explore the aging experiences of older men and women with SCD. The results can be used to identify important health areas for managing older adults with SCD and generate new knowledge useful for enhancing the standard of care for older adults with SCD.

## MATERIALS AND METHODS

### Research design and participants

This study used a qualitative descriptive design, using purposive sampling.^15^ Our sample included older adults with SCD (confirmed via laboratory diagnosis), aged 50 years and older, who spoke English (via self-report). We excluded individuals who were previously diagnosed with moderate-to-severe cognitive impairment, unable to provide informed consent, or wheelchair-bound which is the same criteria as the parent study that was focused on developing a functional assessment of older adults with SCD.^8^ We recruited participants from a single academic center in the Southeastern United States. For the parent study, 44 of 55 adults approached agreed to participate. Of the 20 older adults (age ≥ 50) approached for this qualitative study, only 1 person declined due to an upcoming joint surgery. This study was approved by the participating institutional review board (IRB# Pro00100358). All study participants provided written informed consent. Consent was obtained by the first author.

It is important to note that all participants were born before 1970, during the Civil Rights Movement, a period before widespread integration was established. At that time, the median life expectancy for individuals with SCD was just 14 years, and no preventative treatments were available.

### Data collection

Participants were first asked to record their information in an electronic survey, which included items on demographics (age, sex, race/ethnicity, etc), social history (education, income, employment status, etc), complications from SCD (avascular necrosis, retinopathy, chronic kidney disease, etc), and non-SCD comorbidities (hypertension, diabetes, heart failure, etc) in a RedCap database using consensus measures from the Phenotypes and eXposures (PhenX) Toolkit.^16^ Participants’ SCD genotype and SCD complications were also extracted from the electronic health record.

**Figure 1.**
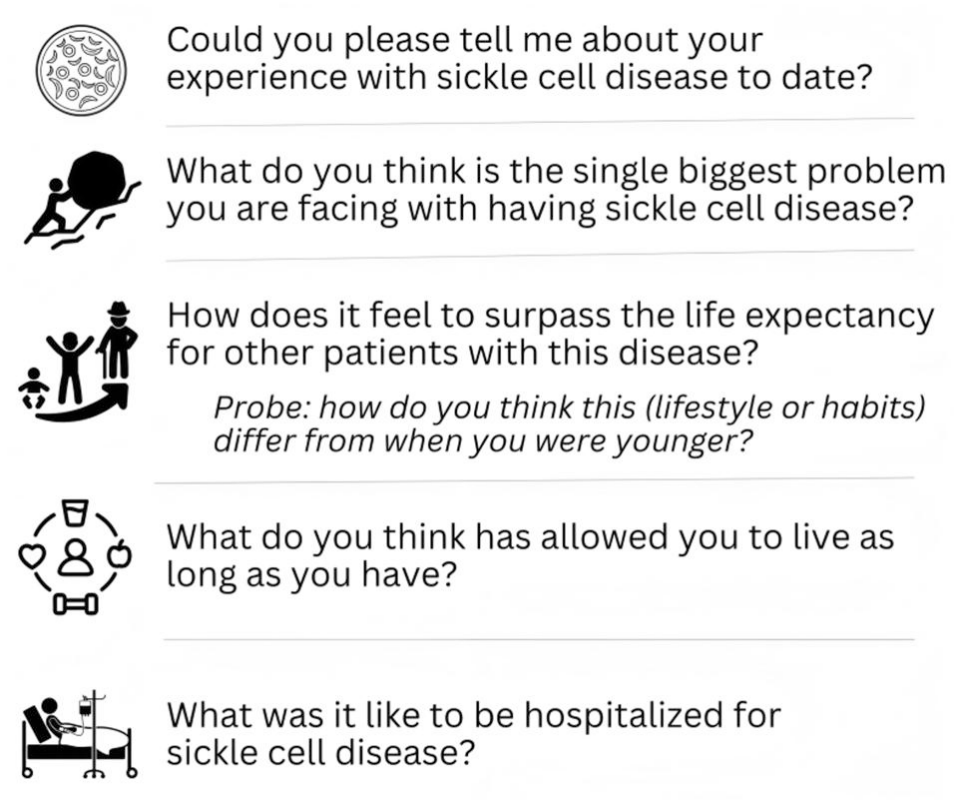
Interview guide sample questions on experiences of older adults with SCD.

The first author conducted semi-structured interviews from June to November 2019 by telephone or in-person at the recruitment site. The interview guide included 18 broad, open-ended questions with specific probes. Interview questions focused on (1) the participants’ experience living with SCD, (2) the biggest challenges participants faced as older adults living with SCD, (3) living beyond the SCD life expectancy, and (4) advance care planning. Sample interview questions are in Figure 1. The duration of the interviews ranged between 30 and 114 minutes. All interviews were audio-recorded and transcribed verbatim by a third-party transcription service. Prior to analysis, all transcripts were edited for accuracy against the audio recording.

### Analytic procedures

We used conventional content analysis to analyze the data because this analytic technique is sufficient to use when little is known about the topic of study, which in this case is the aging experiences of older adults with SCD.^17^ Conventional content analysis has 3 phases: preparation, organization, and reporting.^17^^,^^18^

#### Preparation

We started exploring the data by having the first author read through each transcript to get a sense of the data. The first author summarized each transcript and documented an impression of the interview, which included notes about responses to interview questions, emotions conveyed, and remarkable quotations and stories.

#### Organization

We began the organization phase of analysis by open coding. We analyzed the data line-by-line to remain as close to the data as possible. We used Microsoft Word to manage the analysis. The first author analyzed all data for the latent (symbolic) and manifest (literal) content using descriptive and in vivo codes written in a column beside the transcript.^19^ After coding each transcript, we added codes into a codebook, with code names, descriptions, and quotation exemplars. After coding the first 5 transcripts, we revised the codebook by merging redundant codes.^19^ Next, we grouped codes into subcategories and categories based on similarities. We updated the definitions for the revised codes, subcategories, and categories to ensure the content of each group was appropriately represented. We recoded the initial 5 transcripts with the revised codes and the remaining 14 transcripts were coded using the revised codebook as well.^19^ We added new emerging codes to the codebook until saturation was reached, where no new codes emerged.^20^ Twelve percent of the transcripts pages were jointly coded by a qualitative expert (second author) and the first author. The third and fourth authors also independently analyzed participant quotes to validate the accuracy of the findings. The codebook was reviewed by all authors to evaluate themes related to the aging experience. We discussed discrepancies in codes until a consensus was reached.

#### Reporting

For reporting, we developed a conceptual framework based on the relationship between the themes and subthemes and included quotation exemplars as evidence of findings.^17^

#### Rigor

To strengthen the rigor of this work, throughout the analysis process we ensured the dependability, credibility, confirmability, and transferability were upheld.^19^ For reproducibility, we kept an audit trail of our analytic process, codes, and themes. For credibility, we included quotation exemplars from most of our participants representing a wide range of experiences as evidence of each theme. For confirmability, we used peer debriefing to ensure interpretations of the data matched what participants shared and an expert in qualitative methods reviewed our codebook multiple times. We also engaged in memo writing to document reflections and insights during data collection and analysis. For transferability, which is how the data can be applied to other contexts, we provided a description of our study participants, sample interview questions, and the context (location and time period) of the interviews.

#### Positionality and reflexivity

We have included a positionality statement to contextualize how the authors relate to the study participants and subject matter and acknowledge the lens through which we interpreted the data. The first 3 authors performed most of the data analysis and identify as Black. The first and second authors are Christian. The first 5 authors are women. The first author who conducted the interviews and the last author are both physicians with expertise in SCD care. The first author is familiar with the study participants through clinical care in the sickle cell center and research in the parent study; however, she was a hematology fellow at the time the interviews were conducted. The first author has multiple family members with SCD, and the third author has SCD. The second author is a qualitative research expert with a nursing background and never met the study participants. We engaged in collaborative reflexivity through frequent open dialogue among the first 4 authors in which we acknowledged assumptions and discussed interpretation of participant quotes and descriptions of themes during analysis and writing. We made every effort to remain as close to the participants words as possible and not overinterpret the data based on our lived experiences as medical professionals or our intersectional racial, class, and gender identities.

## RESULTS

The mean age of the 19 participants was 58 years (range of 50-71). Most participants identified as African American (n = 18), male (n = 10), and had an annual household income of ≤$50 000 (n = 10). Almost half (n = 9) were working full- or part-time, and the remainder were disabled (n = 6), retired (n = 2), or unemployed/other (n = 2). Most participants (n = 15) had greater than a high school education (Table 1). Over half had a severe SCD genotype (HbSS, n = 10) and the remainder had HbSC or Hb Sβ^+^ thalassemia. Participant disease characteristics and therapeutics are described in Table 2.

**Table 1.** Demographics and social history of study participants.

Characteristics	Participant (n = 19)
Mean age, years (range)	58 (50-71)
	**n (%) **
Female	9 (47%)
Race	
Black/African American	18 (95%)
Other, Israeli	1 (5%)
Household Income/year	
< $10 000	2 (11%)
$10 000-$50 000	8 (42%)
> $50 001	8 (42%)
Refused to answer	1 (5%)
Employment status	
Working	9 (47%)
Unemployed	1 (5%)
Disabled	6 (32%)
Retired	2 (11%)
Other	1 (5%)
Education level	
High school graduate or less	5 (21%)
Some college/associates/technical school	7 (37%)
Bachelor’s degree	5 (26%)
Advanced degree	3 (16%)
Household size	
1	4 (21%)
2	9 (47%)
3	4 (21%)
≥ 4	2 (11%)
Marital status	
Married/domestic partner	12 (63%)
Single/never married	3 (16%)
Divorced/separated	4 (21%)

**Table 2.** Disease characteristics and therapeutics for participants.

Disease characteristics	(n = 19)
	**n (%) **
SCD genotype	
Hb SS	10 (53%)
Hb Sβ^+^	2 (11%)
Hb SC	7 (37%)
Avascular necrosis	15 (79%)
Joint surgeries	6 (32%)
Retinopathy	11 (58%)
Chronic kidney disease	9 (47%)
Pulmonary hypertension	5 (26%)
Heart failure	3 (16%)
Stroke	2 (11%)
Cutaneous leg ulcers	3 (16%)
Hypertension	7 (37%)
Diabetes mellitus	1 (5%)
Percentage hospitalized in the last year	9 (47%)
Percentage with severe pain crises at home not requiring medical attention/6 months	14 (74%)
	**Median (interquartile range)**
Hb (g/dL)	8.9 (6.5-11.1)
Fetal Hb (%**)**	4.9 (0.9-15.7)
**Therapeutics/medications **	
	**n (%) **
Hydroxyurea	11 (58%)
Chronic transfusion therapy	3 (16%)
Folic acid	14 (74%)
Iron chelation therapy	3 (16%)
Short-acting opioids	16 (84%)
Long-acting opioids	7 (37%)

**Figure 2.**
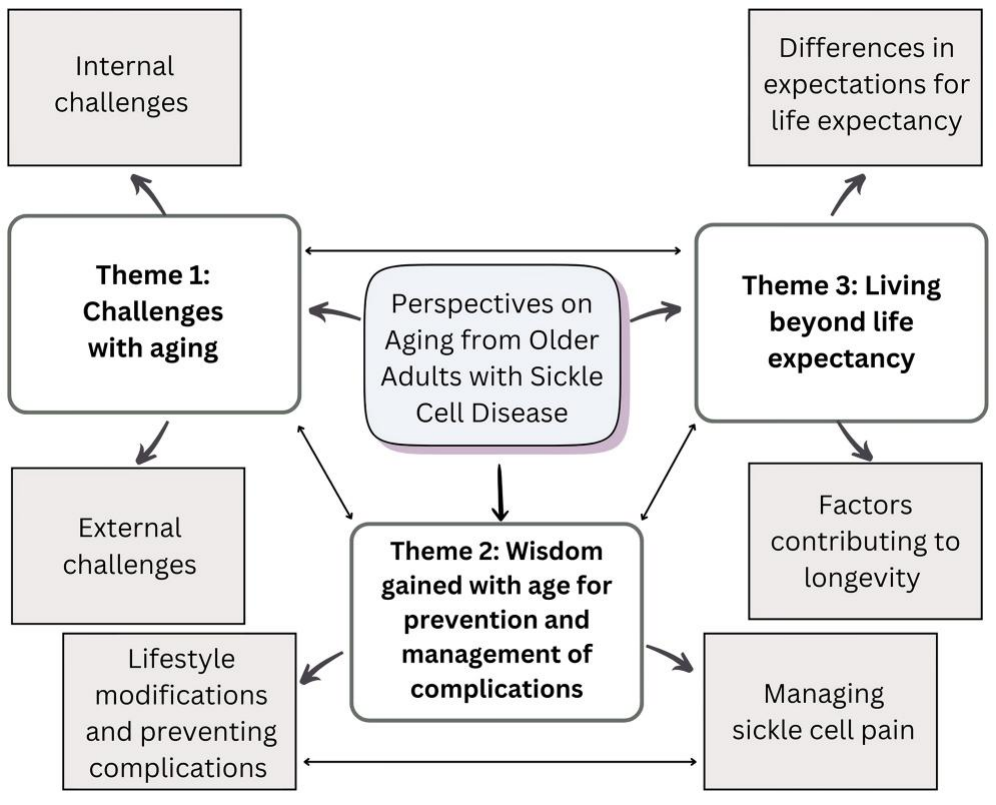
Conceptual framework describing themes and subthemes on perspectives on aging from older adults with SCD.

There were 3 major themes that were identified from the data that describe experiences with aging in older adults with SCD: (1) Theme 1 was “challenges with aging”; (2) Theme 2 was “wisdom gained with age for prevention and management of complications”; and (3) Theme 3 was “living beyond life expectancy.” The conceptual framework describing the major themes and subthemes is illustrated in Figure 2.

### Theme 1: challenges with aging

Theme 1 was “challenges with aging,” which included descriptions of challenges participants experienced across their lifespan. The subthemes in this theme were (a) internal challenges and (b) external challenges. When participants were asked about the single biggest problem they faced as an older adult with SCD, they described challenges from multiple sources. Participants described internal challenges such as physical limitations and fatigue that made them unable to do as much as they perceived others without SCD can do and how pain and SCD comorbidities interfered with their ability to work and travel. Participants also described external challenges with healthcare systems and the workplace.

#### Subtheme I: internal challenges

In this subtheme participants described physical, emotional, and psychological challenges they experienced from childhood to the present. They also described changes in their symptoms, pain experience, and comorbidities that occurred with aging.

**Symptoms and co-morbidities**. Physical limitations were the most common challenges older adults with SCD described. These physical limitations had a variety of causes including pain, fatigue, SCD comorbidities, and mental health issues. One participant described his experience with physical limitations:

The limitations that it's placed on me being physical. I'm not going to be able to do what other people can do … say like when I wanted to get me a vehicle. Got me a job and started working and wanted to save my money, and then, within 6 months to a year, I'm sick with pneumonia or a sickle cell crisis. So as that kept repeating itself through life, it kind of lowers your wish list in life because you know some things you're just not going to be able to achieve because of the limitations that sickle cell has placed in your life. (FGA007, 63-year-old male, HbSC)

Some participants felt like fatigue was one of the biggest challenges they were dealing with as an older adult, “Well, at this point, the worst thing for me is still dealing with this feeling of chronic fatigue. That's affected me more and on a consistent and daily basis more so than anything else” (FGA020, 62-year-old female, HbSC).

They described SCD complications such as pulmonary hypertension, recurrent sickle cell pain crises, organ failure, and chronic leg ulcers that limited their function so much that some participants had to retire early or develop new skills to work different jobs that suited their current physical abilities. They often described how physical limitations caused by pain, fatigue, and stiffness made it difficult to do activities they enjoyed such as dancing or traveling long-distances by car.

Some participants discussed how pain and fatigue worsened as they got older. They described how their recovery time after a sickle cell pain crisis was also slower compared to when they were younger. Despite these changes, many participants were determined to press on through these challenges. A participant discussing how they persevere stated:

But as I get a little older, the symptoms and the problems may get a little worse—I understand that—but in my mind, I can't let it stop me from doing anything that I need to do. (FGA003, 55-year-old male, HbSS)

In contrast, there were some participants that noted improvements in their pain crises as they got older. A participant described how going through menopause improved her pain. She stated:

And then I went through ‘The Change.’ I think that helped a lot … Because that's when I stopped having a period. Menstruating. So then I started having fewer and fewer crises. When they came, they still were very painful. But it was fewer. And I started taking care of myself at home. I didn't go to the emergency. I didn't go to the hospital. It's been over 20 years since I've been hospitalized for a crisis. (FGA011, 71-year-old female, HbSS)

Participants described how having SCD affects their mental health, and how sometimes the mental and emotional challenges triggered onset pain episodes, and at other times the pain episodes caused mental and emotional challenges. A participant described her experience with interwoven pain and mental health challenges shared, “It used to be very overcoming and would lead me to bouts of depression at times and I will have a crisis” (FGA008, 52-year-old female, HbSC).

#### Subtheme II: external challenges

Participants described a variety of challenges they experienced from external sources such as in the workplace, people in healthcare settings, and from friends and family.

Challenges in the workplace were very common. Participants described how employers were not always understanding of their health condition and did not always accommodate requests for time off to recover from a pain crisis. When describing his experience of being asked to work after being recently hospitalized a participant shared:

I would stay in the hospital for like 2 days, maybe 3 days, get out, but then they'll expect for me to be at work. And that's the biggest thing I just don't understand. And sometime I did it, sometime I didn't. And because sometime I could, sometime I couldn't. (FGA003, 55-year-old male, HbSS)

Participants described difficulties maintaining jobs due to frequently missing work. Some participants discussed how they felt forced to retire early or go on disability due to external pressure from their healthcare provider and/or employer even when their preference was to continue working. Multiple participants described how as they developed more health issues, they had difficulty maintaining employment or operating a business. Some participants described having to develop new skills and work a different job that was easier on their body. One participant described a time when he had to stop working due to complications of SCD which led to depression; however, he was determined to move forward and figured out what he could do:

I guess I've always been determined not to let something whoop me. And I did find myself sitting at home one time dying. And I had to talk to myself, you know, that you can't just sit—I mean, when I found out what I had, I was depressed, yes. And I thought that, well, I can't do nothing. I can't do this no more; I can't do that no more. And one day I just had a change of heart and I told myself, ‘You can't do this. You got to move on.’ And I looked at other people with disabilities and they don't let it stop them, so I said, well, if they can do it, I can do it. Only thing is, I just got to control. (FGA010, 62-year-old male, HbSC)

Another participant described having conversations with his physician on when to retire from his business due the severity of his leg ulcer:

I mean, I started having these ulcers in my leg, and that's when it really started getting bad. My doctor asked me if I was ready to retire yet, and I told him, ‘No.’ So I went another 4 or 5 years before I even thought about it because I was starting to get bad ulcers. I had to stay out of work couple weeks and go back a week and so it got to be annoying with the job. So he took me out of work and that's where I am today. (FGA009, 63-year-old male, HbSS)

Participants described challenges related to perceived stigmatization from colleagues and teachers. They discussed the negative impacts of encountering people who express misconceptions about SCD and even at times have had experience where colleagues did not believe that they have SCD. A participant described his experience being sick in school and being mistreated by a professor because of his disease. This experience made him feel ashamed to share his SCD diagnosis with others:

When I was in college, and I got sick—it was funny because one of the professors walked up to me and say, ‘You know, you sho’ be out for a long time sometime. If you have AIDS you need to get some help.’ And I just looked at her and I told her. I said, ‘Well, you’re wrong about the diagnosis.’ I say ‘but I’m pretty sure you’re not going to believe unless I tell you why I was out.’ So once I told her I had sickle cell it was funny because she didn’t have nothing else to say. She just looked at me and turned her head and walked away, so. (FGA001, 54-year-old male, HbSS)

**Figure 3.**
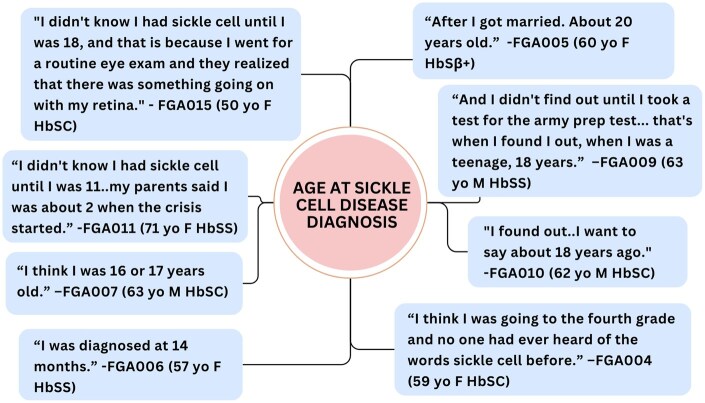
Descriptions of delayed diagnosis of SCD and the age at diagnosis. Participants discussed their experience being diagnosed with SCD several years after birth, with some people even finding out as late as their 40s. This is a unique experience for older adults with SCD since newborn screening was not yet standardized in the United States when they were born. This figure contains quotation exemplars of participants describing their age and the circumstances of when they first found out they had SCD.

Participants described several challenges in healthcare settings. Many of the challenges they faced were due to limited healthcare provider knowledge on caring for people with SCD, a lack of clinical practice guidelines for SCD when they were growing up, and a lack of standard newborn screening protocols which led to delayed SCD diagnoses. Participants frequently reported being diagnosed with SCD several years after birth. They described living for many years undiagnosed, not knowing the cause of their symptoms and how best to treat their illness. Several participants expressed frustration with healthcare providers for being dismissive of their pain and undertreating their symptoms. Figure 3 describes the age and how participants reported being diagnosed with SCD.

A participant discussed the need for more treatments and providers who are knowledgeable about caring for older adults with SCD:

They’re going to be more people living as long as I am or even longer. And there gon’ to have to be some more medicine popping up somewhere. Cuz I’m tired walking into a doctor’s office asking me ‘why I’m still here, I’m not supposed to be here.’ And that’s telling me you don’t know your job. So you got to go back in the books to come up, to catch up with me. I’m living. You hate that because you got to work. (FGA002, 67-year-old male, HbSS)

Several participants also discussed being stigmatized by healthcare providers as a “drug-seeker,” being misdiagnosed and sent home too soon. A participant described how different the healthcare system was and challenges with being stigmatized and getting inadequate care:

It was much different than it is today because … pain medication was more restrictive. You took pain medication every 4 to 6 hours no matter if the crisis came back, no matter how severe the pain, that was it … I hated going to the hospital. And those chairs were so so hard. And I just hated it because you had to sit there and wait and I’m in pain. You don’t get any pain medication. So I really hated it … Like I said, you had to wait no matter—and then sometimes the doctors’ attitudes were—. You were many times labeled as a junky coming to get your fix. But speaking about the pain medication was your fix, and you weren’t really viewed as someone who was really having the pain. Not with every hospital and every doctor, but you did run into that. (FGA011, 71-year-old female, HbSS)

They also reported life-threatening complications they experienced because of providers not knowing how to properly care for them and described their fears and frustrations surrounding healthcare providers not listening to them. One participant described her experience being in a hospital, “Again, you are kind of at the mercy of people who have limited knowledge of treating you or what they’re treating. And then often times they don’t listen” (FGA016, 50-year-old female, HbSC).

### Theme 2: wisdom gained with age for prevention and management of complications

Theme 2 was “wisdom gained with age for prevention and management of complications.” This theme included descriptions of knowledge participants acquired over the years that helped them to prevent and cope with SCD complications. They discussed lifestyle modifications and strategies that they have used to treat and recover from sickle cell pain crises. Several participants described the overall importance of having a positive outlook and determined mindset as a person living with SCD. Subthemes related to prevention and self-management were (a) lifestyle modifications and preventing complications and (b) managing sickle cell pain. Participants made a distinction between strategies they used to prevent pain crises from occurring versus strategies they used to manage pain crises once they were already in a pain crisis and were trying to recover. There often was overlap between the prevention and management strategies such as physical activity and hydration. However, there were differences in how they used the strategy to prevent a pain crisis versus recover from a pain crisis (ie, doing light walking or gentle movement to recover from a pain crisis versus consistent exercise to prevent pain crises).

#### Subtheme I: lifestyle modifications and preventing complications

Participants described positive habits to prevent sickle cell pain and other complications of SCD, such as how they avoided certain environmental conditions and unhealthy habits. They emphasized the importance of knowing triggers of pain and actively working to avoid them. Several people discussed how, as they got older, they learned to prepare with plenty of water and appropriate clothes for weather extremes when they went out. Participants discussed how a healthy diet and consistent exercise helped them have fewer pain crises. Some participants sought advice from a physical therapist on how to remain active without exacerbating pain related to avascular necrosis of the joints. Some described how remaining active with work made them happy and reduced their hospital admissions. A participant described how she learned to care for herself better as she got older:

And during that time, being in my 20s, I was just working like crazy, hanging out with friends and not being mindful of staying well hydrated, not getting over-stressed and just was not resting. And as I've gotten older and wiser, I've learned, ‘Okay, these are my triggers. If I get low, this is how I need to take care of myself.’ And I have to really care for myself so that I do not go into a crisis. So I think … educating myself … and learning to truly not overdo it in my life. (FGA015, 50-year-old female, HbSC)

Several participants described the importance of maintaining good physical and mental health to remain healthy. A participant discussed the connection between his mental health and body. He expressed the importance of remaining positive and stress-free, stating:

And I try to exercise and be healthy — … a lot of that also is trying to keep a positive attitude and not another frame of mind. And to relieve stress as much as possible. So that doesn't impact my physical well-being. (FGA017, 52-year-old male, HbSS)

In addition to avoiding stressful situations, participants avoided specific exposures such as dehydration, extreme cold (indoors or outdoors), processed foods, drugs, alcohol, and other substances that could negatively impact their health. One participant shared the negative habits he avoided and positive habits he did to remain healthy, “I've never smoked. I've never drank, never did no street drug or anything like that—I've always been very active. Walking around, get plenty of exercise …” (FGA009, 63-year-old male, HbSS).

Participants also emphasized the importance of knowing their limits in order to prevent sickle cell pain. They described how their experiences with overexertion to the point of triggering pain taught them their physical limits and how to avoid going beyond their limits. Physical exertion most frequently occurred while they were working. They had to modify their activity at work when engaging in movement patterns or activities that could trigger pain. Some participants had to learn new skills so they could do a different job that was not as hard on their body:

I had to find out how I could work. And know when not to or when to back off. So I had to teach myself how to be able to work. And I could feel a crisis, which I didn't know when to start with, that's when I found out, okay, I can work. But it's a limit that I can do. So once I reach that limit, I back away. (FGA010, 62-year-old male, HbSC)

#### Subtheme II: managing sickle cell pain

Participants shared strategies that they have used to manage sickle cell pain without direct intervention from healthcare providers. Many of the strategies used to prevent SCD complications are also used to self-manage and help recover from SCD pain episodes.

One participant discussed various strategies she employed simultaneously at home to cope with sickle cell pain. She discussed both pharmacologic and non-pharmacologic strategies:

I've learned how to take my meds, if I need time out I find me a quiet place which is usually my bedroom, I drink plenty of liquids and try to sleep it over and pray through it and meditate through it. (FGA008, 52-year-old female, HbSC)

Participants described helpful non-pharmacologic pain management strategies such as meditation, aquatic therapy, physical therapy, and acupuncture. Some discussed how they had to advocate for themselves to receive services like physical therapy. Alternative pain management strategies were not readily offered by their healthcare providers. This person described her experience, “… it [my blood] kind of thinned it out after I had acupuncture. I went and had acupuncture, and it seemed like my blood flowed better” (FGA005, 60-year-old female, HbSβ^+^).

Not only did physical activity prevent SCD pain episodes but some participants noted that physical activity like walking during a pain episode also helped them to recover from sickle cell pain. One participant described how walking helped him recover during a pain episode, “Always, when I got halfway to feeling good, I start walking around the units so that way I can get back to normal” (FGA019, 58-year-old male, HbSβ^+^).

Overall participants described the wisdom they have acquired over a lifetime of living with SCD that allowed them to deal with sickle cell pain more efficiently, despite the new challenges of aging.

### Theme 3: living beyond life expectancy

Theme 3 was “living beyond life expectancy” which included descriptions of their experience and attitudes on living beyond age 50 with SCD. They detailed their perspective on which factors allowed them to surpass the life expectancy for SCD. They described how their expectations for lifespan were both positively and negatively influenced by healthcare providers and family members. Subthemes related to living beyond life expectancy were (a) differences in expectations for life expectancy and (b) factors contributing to longevity.

#### Subtheme I: differences in expectations for life expectancy

Participants described differences in their expectations for how long they would live. Despite several participants being told that they would not live into adulthood, many of them still believed that they would live a long life. They described how their faith and family encouraged them to believe they would survive beyond the time of their given prognosis. A participant describing their will to live stated:

I just believed that I was not gonna die. I just believed that I was going to live out my life. I don't know. I don't know. Grace. It's got to be the grace of God that I've lived this long. (FGA004, 59-year-old female, HbSC)

Some participants who were told they would not live long avoided making plans for the future. For example, one participant shared how expecting to die before adulthood made him reluctant to make future plans; however, he began making plans once he surpassed the age he was told he would die:

So they were saying I would never make it to 20. So that's when I decided okay I'm not getting married. I'm not going to buy a house. I'm not going to do any of that stuff. I'm going to go to work, and I'm just going to spend my money and have fun while I'm doing it. And that's what I did until I got 21 and like hold on, wait a minute, I done passed by 20 now [laughter]. So I said, ‘Well, maybe I may need to save a little money. I may need to buy me a house or something.’ So that's what I end up doing. (FGA001, 54-year-old male, HbSS)

A participant described how it felt good to live far longer than she originally thought instead of dying early:

Well, I never thought I'd live this long because years ago the life expectancy—my life expectancy was 30s, so I never thought I would live to be 72. So it feels good when you think about what the other choice is. (FGA011, 71-year-old female, HbSS)

This subtheme is interconnected with the “external challenges” subtheme of theme 1, “challenges with aging,” since several participants were told by healthcare providers that they would not survive into adulthood.

#### Subtheme II: factors contributing to longevity

Participants discussed several factors that they felt contributed to them living longer. The most common response was that they believed it was God who allowed them to live as long as they have, as one participant stated, “Well, my theory is, it's the good Lord” (FGA012, 61-year-old male, HbSS).

Participants also felt they lived longer because they ate a healthy diet high in fruits and vegetables with limited sugar and salt, exercised frequently, engaged in activities and work that made them happy, were diligent about their healthcare (eg, attending all their doctor’s appointments, and focusing on preventive strategies with their healthcare providers), had genetic factors that promoted a longer life, and were determined to live. A participant described how being proactive has contributed to her longevity:

I think being very diligent about my healthcare has been one and being a part and not like living on the outside. Being very focused about trying to live a healthy life and doing a lot of research when I was just young about sickle cell and what my expectations should be and those things that I should be doing, those things that I shouldn't be doing. Not that I always did everything right but having that knowledge. Therefore, when I went to a doctor appointment, and I would have questions and brought up things that we can talk about so we would be doing the preventive things as well as trying to take care of whatever crisis was going on at that moment and having good healthcare providers who allowed that who understood that that was necessary and who encouraged that. (FGA006, 57-year-old female, HbSS)

They described a determined mindset that helped them to overcome challenges and continue to live. Despite difficulties related to their health and knowledge of a possibly limited life expectancy, participants focused more of their attention on finding satisfaction and living the fullest life possible:

If I were to die tomorrow, I think I would have lived my best life without a question, because I was never denied or restricted anything. And, I've done everything I possibly want to do, I possibly could've done and can do 2 or 3 times over. (FGA018, 51-year-old female, HbSS)

## DISCUSSION

The purpose of this study was to describe aging experiences of older adults with SCD including their single biggest challenge, prevention and management strategies that have contributed to their survival, and unique experiences living beyond the life expectancy for SCD. Key findings of this study are that older adults have been determined and resilient while overcoming personal and societal challenges. The wisdom they acquired to overcome or adapt to these challenges and the lessons they have learned about preventing and self-managing SCD complications may serve as a guide for others aging with SCD.

The findings of this study significantly contribute to the literature by providing deep insights into the lived experiences of older men and women living with SCD. New insights uncovered in this study include the biggest challenges older adults with SCD face (ie, difficulties working, maintaining employment, and limitations in physical function), how this population navigated aging and making it to adulthood after being told they would die, and strategies they felt helped them to exceed life expectancy despite not having a blueprint or evidence-based medical guidance. By capturing personal narratives, this study enriches our understanding of the unique perspectives of this population that have not been historically captured by quantitative methods

Several participants described challenges with physical limitations and accessing pain management (theme #1) previously described by Jenerette et al.^14^ Some of the challenges are similar to those experienced by older adults without SCD, specifically concerns about their ability to function day-to-day and pain management. In the Baltimore Study on Black Aging, men and women who reported more pain had greater disability in their activities of daily living compared to those without pain. African American women with 2 or more comorbid conditions were significantly more likely to have impaired activities of daily living.^21^

Several of our participants described the single biggest problem (theme #1) they faced was maintaining employment and/or challenges in the workplace. They acknowledged the importance of being able to work, but some had difficulty maintaining employment due to missed workdays dealing with health issues, while others had to retire early. An international systematic review on the health effects of working as an older adult (age > 64 years) showed beneficial or neutral effects as long as the work was not a high demand or low reward job.^22^ Unemployment is associated with increased pain frequency in adults with SCD.^23^ The Sickle Cell World Assessment Survey showed that people with SCD in the United States experience greater impact from SCD on employment compared to other high-income and low-to-middle income countries. These negative impacts included challenges with finding a suitable job, attending work, keeping a job, and needing disability or long-term sick leave.^24^ There are minimal data on vocational rehabilitation and other programs to increase employment for adults with SCD. Vocational rehabilitation has increased employment in disabled individuals in Europe and Canada.^25^^,^^26^ Specifically programs that include work-focused cognitive behavioral therapy, multidisciplinary interventions, and individual placement and support have been successful in allowing individuals to return to work.^25^

The participants in this study grew up during a time when Jim Crow laws were being overturned and public facilities such as hospitals, schools, and places of work were being integrated due to the landmark Civil Rights Act of 1964, which prohibited discrimination based on color, race, sex, religion, and national origin. In addition, they also grew up during desegregation of neighborhoods due to the Civil Rights Act of 1968 prohibiting housing discrimination. Despite these efforts, structural racism has still been pervasive in the United States, limiting access to quality healthcare, employment, and financial opportunities for Black people.^27^ Moreover, even in modern times there are several studies showing racial bias in the United States. Healthcare system that manifests as false beliefs about biological differences between Black and White people leading to Black people being systematically undertreated for pain.^28^^,^^29^ Our study participants did not overtly discuss race as a driving factor for the inequities they experienced in the healthcare system and in society. Participants mainly described inequities such as undertreatment of pain and discrimination at school or work as stigmatization based on having SCD. This is consistent with prior studies on stigma in people SCD that show that people with SCD perceive a greater amount of disease-based discrimination compared to race-based discrimination and have a greater burden of perceived race-based discrimination compared to other Black people and all other races.^30^ In addition, older age has been associated with greater perceived race-based discrimination.^30^ Jenerette et al. described how disease-based stigma for people with SCD begins during childhood.^31^ Disease-based stigma can have a negative impact on the health of people with SCD and is associated with depression, a higher burden of SCD pain, and can hinder them seeking appropriate care for acute pain episodes.^30–32^ Improving provider communication may decrease stigma during healthcare encounters.^33^

Other studies in Black people without SCD identified similar themes especially in regards to external challenges (theme #1) and self-management strategies (theme#2).^34^ A study of older Black women (age ≥ 50 years) showed that participants perceived their healthcare providers did not understand their pain and that they had to push themselves physically and mentally to function despite having pain.^34^ They also described the benefits of non-pharmacologic strategies for managing pain such as exercise, yoga, water therapy, massage, and meditation.^34^

Participants in this study described high levels of self-care abilities and self-efficacy (theme #2). Higher self-efficacy, which is defined as a person’s belief in their ability to control life events and behaviors, is associated with increasing knowledge, spirituality, and maternal care during childhood.^35–38^ Higher disease self-efficacy is associated with better health-related quality of life and physical and psychological symptoms.^35^^,^^39^^,^^40^ Studies in young adults with SCD have shown that self-efficacy is independent of age and gender,^37^ and chronic pain and disability were higher in older groups (age 31-40 years) compared individuals age 18-25 years.^41^ It is unknown how self-efficacy changes in adults with SCD over the age of 40.

Faith in God and healthy lifestyle choices were described as factors that contributed to longevity in older adults with SCD (theme #3). There is evidence that supports our participants’ notion that their healthy lifestyle choices have contributed to their longevity. Although individuals in the United States have a lower life expectancy compared to other high-income countries, life expectancy could be prolonged by adopting 5 low-risk habits: never smoking, maintaining a healthy diet and weight, regular physical activity, and limited alcohol consumption.^42^

Similar to community-dwelling older adults, our participants identified their mindset as a major factor contributing to their longevity. A qualitative study of interviews about successful aging with community-dwelling older adults identified 2 main themes: (1) the importance of achieving self-acceptance/self-contentment and (2) engagement with life/self-growth.^43^ Our participants also described self-contentment with work as being important and having a positive and determined outlook as key components for successful aging.

There are several factors that are unique for people aging with SCD as opposed to the general aging population. People with SCD experience unique disease-related impacts that are not typically seen in geriatric populations such as a vaso-occlusive pain events, avascular necrosis of the joints often requiring joint surgery at a younger age, a weaker immune system related to functional asplenia or splenectomy, leg ulcers, sickle cell retinopathy, pulmonary hypertension, and a shorter life expectancy.^6^ Aging with SCD is also unique because they have to consider going on disability or retirement at a younger age compared to the general population.^24^ They also have to consider advance care planning earlier in life.^18^ They must face their own disease-related and age-related challenges during a time when some individuals are in the sandwich generation having to simultaneously raise their children and care for their aging parents. Older adults with SCD also have limited dedicated frailty and functional assessment and management resources in contrast to robust assessments and interventions for the geriatric population to help preserve their independence and improve function.^8^

### Strengths and limitations

The main limitation of this study is recruitment from a single site, a large academic center in the Southeastern United States. Our research participants were probably more engaged in their healthcare compared to the general population of people with SCD as they were willing to participate in this research and routinely obtained their care at a comprehensive sickle cell center. To better understand a wider range of experiences with aging as an older adult with SCD, a multi-regional qualitative study would need to be conducted. Another limitation was that the same researcher conducted interviews and analyzed most of the interview transcripts. This creates a possibility of bias; however, we sought to minimize this bias by having 2 additional research team members review all quotes independently and then we discussed the quotes as a group until we reached a consensus on the themes. In addition, there were many other important experiences only briefly explored in these interviews, such as pain, relationships/social support, and navigating the healthcare system as an older adult with SCD that deserve to be explored further in future interviews.

This study has several strengths since it is the largest study to date describing the aging experience of older men and women with SCD in the United States. This study focuses on the most pressing challenges this population faces in modern time. Participants also provided practical and actionable advice on how they have managed to prevent pain and complications in different stages of their lives. This advice emphasized the importance of access to non-pharmacological interventions to treat pain and improve function as well as programs to support employment for people with SCD.

## CONCLUSION

Older adults offer valuable insights gained from their experiences living and aging with SCD. Their knowledge about prevention and management of SCD complications and factors that contribute to living beyond life expectancy will guide research and interventions aimed at promoting successful aging with SCD. This information will help shape peer mentoring programs for younger and middle-aged adults with SCD to improve their self-efficacy. Additionally, it will support the development of initiatives that directly address the biggest challenges faced by older adults with SCD by utilizing sickle cell-specific geriatric assessment and management tools.

## Data Availability

Data are not available because raw participant interview data cannot be shared due to ethical restrictions.
